# Red-lesion extraction in retinal fundus images by directional intensity changes’ analysis

**DOI:** 10.1038/s41598-021-97649-x

**Published:** 2021-09-14

**Authors:** Maryam Monemian, Hossein Rabbani

**Affiliations:** School of Advanced Technologies in Medicine, Medical Image & Signal Processing Research Center, Isfahan University of Medical Sciences, 8174673461 Isfahan, Iran

**Keywords:** Imaging and sensing, Diagnostic markers, Retinal diseases

## Abstract

Diabetic retinopathy (DR) is an important retinal disease threatening people with the long diabetic history. Blood leakage in retina leads to the formation of red lesions in retina the analysis of which is helpful in the determination of severity of disease. In this paper, a novel red-lesion extraction method is proposed. The new method firstly determines the boundary pixels of blood vessel and red lesions. Then, it determines the distinguishing features of boundary pixels of red-lesions to discriminate them from other boundary pixels. The main point utilized here is that a red lesion can be observed as significant intensity changes in almost all directions in the fundus image. This can be feasible through considering special neighborhood windows around the extracted boundary pixels. The performance of the proposed method has been evaluated for three different datasets including Diaretdb0, Diaretdb1 and Kaggle datasets. It is shown that the method is capable of providing the values of 0.87 and 0.88 for sensitivity and specificity of Diaretdb1, 0.89 and 0.9 for sensitivity and specificity of Diaretdb0, 0.82 and 0.9 for sensitivity and specificity of Kaggle. Also, the proposed method has a time-efficient performance in the red-lesion extraction process.

## Introduction

Diabetic retinopathy (DR), a common disease among the patients with the history of diabetes, is the most important reason for blindness in developed countries. Studies show that approximately 80 percent of patients with 10 years’ history of diabetes are influenced with DR. Currently, 93 million people live with diabetes around the world. The early diagnosis and monitoring of DR can prevent from irreversible damages to human eye. DR is defined as the damage to blood vessels in retina. In fact, the high blood sugar in the diabetic patients can close the tiny blood vessels of retina and lead them to have leakage. In some cases, the eye makes new blood vessels that may not work well^1^.

Fundus imaging is defined as the process by which a 2 dimensional representation of the 3 dimensional tissue is achieved with the help of reflected eye. With respect to the significant increment in the prevalence of DR especially between aging population, the necessity of retinal examination becomes more important^2–24^. Fundus images are widely utilized for evaluating and grading of DR. Retinal fundus imaging can produce beneficial information about diseases such as DR, macular edema and glaucoma^25^. The symptoms of DR in fundus images are manifested as red lesions including Hemorrhages (HMs) and Micro-aneurysms (MAs) and bright lesions like exudates. The manual verification of such abnormalities in the retinal fundus images is a tedious, time-consuming and error-prone task. Therefore, the design of automatic Computer-Aided Diagnosis methods for the rapid and accurate monitoring of DR is of considerable importance.

In order to evaluate the severity of DR, it is necessary to extract the related retinal abnormalities. However, this process encounters challenges due to the low quality of retinal fundus images, different shapes and sizes for abnormal lesions, similarity between some parts of blood vessels and red-lesions. Many research works focused on suggesting solutions for the mentioned problems. However, long processing time and unnecessary complicated computations have reduced their efficiency. Thus, to propose new methods with high accuracy, simplicity and speed in the detection of abnormalities of retinal fundus images is considerably beneficial to help ophthalmologist in the evaluation of DR.

The methods focused on detecting abnormalities in retinal fundus images can be categorized into mathematical morphology-based^26–29^, region growing-based^30,31^, wavelet-based^32–34^, pixel classification^17^, artificial intelligence and deep learning^35,36^, knowledge-based^37^ and hybrid approaches^38–41^. In the next section, each class is described by explaining sample related research works.

In this paper, a novel red-lesion detection method for retinal fundus images is proposed which provides efficiency in terms of speed and accuracy. This method works based on the detection of all boundary pixels with the help of analysis of intensity changes. It also distinguishes the boundary pixels of red-lesions from the boundary pixels of blood vessels via extracting unique features for red-lesions.

The rest of paper is structured as follows. Section II explains the most important research works in the field of abnormality detection in retinal fundus images. Section III describes the proposed method in details. Section IV includes discussion about the new method and its numerical evaluations. Finally, section V consists of several concluding remarks.

## Literature review

In this section, a number of most important research works which are related to the field of abnormality detection in retinal fundus images are reviewed.

It is possible to categorize the existing methods for abnormality detection in retinal fundus images into mathematical morphology-based^26–29^, region growing-based^30,31^, wavelet-based^32–34^, pixel classification^17^, artificial intelligence and deep learning^35,36^, knowledge-based^37^ and hybrid approaches^38–41^.

In Ref.^17^ a method for detecting red-lesions is proposed which firstly separates vasculature and red-lesions from the background using a pixel classification method. Then, the connected vasculature is identified and removed to specify the red-lesions. However, as mentioned in Ref.^17^ the required time for candidate detection is considerably high in the pixel classification approach.

With respect to the mathematical morphology-based methods, a red-lesion detection and classification method was proposed in Ref.^26^. This method consists of four steps. At first, the green channel of fundus image is extracted due to its better contrast. In the second step, the candidates for red-lesions are segmented using mathematical morphology and phase congruency and the aim is to reduce the number of falsely interpreted regions as red-lesions such as blood vessels. In the third step, MAs and HMs are discriminated according to the features extracted from the candidates in the second step. In the final step, K-Nearest Neighbor (kNN) and Support Vector Machine (SVM) are utilized to label the candidates as true or false. However, there is no need to segment the boundaries of each candidate region for detecting red-lesions. The reason is that some of them may be mistakenly identified as red-lesions and are removed in the next steps. Another red-lesion detection method for fundus images is proposed in Ref.^27^. The method firstly removes optic disc and blood vessels from the image. Then, an edge enhancement method based on curve-let transform is executed to separate out darker spots from background. Also, a band-pass filter is designed to enhance bright lesions. Then, morphology-based post-processing is performed to refine the obtained lesions. In Ref.^28^ a method for extracting exudates from retinal fundus images is proposed. It utilizes morphological operators such as geo-disc erosion, geo-disc dilation, reconstruction by dilation or erosion to determine the contours of exudates. It also detects the optic disc with the help of the mentioned operators and also watershed transformation.

In Ref.^31^ a method is proposed for vessel segmentation in retinal fundus images which works based on local adaptive thresholding. Multiple thresholds are used to produce binary images and the final vessel-segmented image is obtained by combining all the results.

A method for detecting red-lesions and exudates is proposed in Ref.^35^. This method includes several steps. In the first step, pre-processing operations such as normalization are performed to enhance the retinal structures. In the next step, the blood vasculature is segmented and the locations of optic disc and fovea are estimated. Then, the image is decomposed into several layers including dark and bright layers that each layer represents a different structure in retina. Then, several features are extracted from the obtained layers. Finally, a Multi-Perceptron Layer is utilized to discriminate the true lesions from falsely detected candidates^35^. However, the segmentation of blood vasculature and decomposition of image into several layers inflict a significant volume of computations. A method based on deep learning is proposed in Ref.^36^ to detect the early signs of DR, micro-aneurysms. In this method, a Convolutional Neural Network (CNN) is utilized to detect MA and non-MA spots. The training phase of this method includes two stages. Firstly, there is a basic CNN where the normal samples are selected from its output probability map. The selected samples are considered as the inputs for the final CNN which produces the probability of being a MA for each pixel^36^. A red-lesion detection method based on deep learning approach is proposed in Ref.^42^. The deep features learned by a Convolutional Neural Network (CNN) along with hand crafted properties are used to detect red lesions by the help of a random forest classifier. Another hemorrhage detection method based on deep learning is proposed in Ref.^43^ which focuses on the reduction of time spent for training CNN. Various types of abnormal lesions in fundus retinal image are segmented in Ref.^44^ and the severity of DR is determined with machine learning algorithms. In Ref.^37^ a method is proposed to find and fill the exudates in the retinal fundus images so that false positive regions are reduced and vessel segmentation is enhanced. The vessel segmentation then is achieved through some Hessian-based filtering method. The method can be beneficial for vessel segmentation in both the normal and the pathological cases affected by exudates.

A method for detecting micro-aneurysms is proposed in Ref.^32^ which works based on the wavelet transform. In this method, the input image is decomposed into several images called sub-bands using the wavelet transform. Each sub-band contains information at a specific frequency. For instance, high and low frequency sub-bands are related to noise and slow image variations which can help in the omission of non-lesions of image. A window-based approach is utilized where the wavelet transform of image restricted to the window is compared with that of model in different sub-bands. Based on these comparisons, it is determined whether or not a window can be considered as a micro-aneurysm. A method for the diagnosis of retinal lesions is proposed in Ref.^33^. The method works based on the wavelet decomposition of green channel of fundus image followed by Hessian multi-scale analysis. In Ref.^34^ a method is proposed for the segmentation of retinal vessels. This method gives a vessel or non-vessel label to each pixel based on the extracted features from them. Feature vectors include pixels’ intensities and also two-dimensional Morlet wavelet transform which in turn helps in the process of noise filtering from retinal fundus images.

In Ref.^38^ a method for automated MA detection in retinal fundus images is proposed which consists of pre-processing, candidate extraction, feature extraction and classification phases. In the candidate extraction phase, candidates are extracted using peak detection and region growing. After extracting candidate pixels, region growing is performed to grow candidate pixel into the original MA shape. The features extracted in the next phase include local features like shape and intensity features and profile features. However, a large volume of computations should be performed in this method. In Ref.^39^ a method is proposed for the detection of MAs that its main phases are similar to Ref.^38^. Candidate pixels are chosen from pre-processed image and MAs are detected through the intensity analysis along the lines segments centered at the candidate pixels. A method for extracting red-lesions from retinal fundus images is suggested in Ref.^40^. After pre-processing, the optic disc is detected and discarded from the image to not include any red lesion candidate. Then, all the red-lesion candidates are detected based on their contrast and intensity features. Also, dynamic shape features are extracted for the candidates. Then, a classifier is utilized to label the extracted candidates. A method for detecting and classifying abnormalities in retinal fundus images is presented in Ref.^41^. In this method a blob-ness measure is proposed and several intensity and shape features are utilized to identify DR related changes. Then, a Support Vector Machine (SVM) is used to classify the abnormal changes of retina.

With respect to the existing datasets for retinal fundus images, it is possible to mention MESSIDOR, DRIVE, STARE, ROC, DIARETDB0, e-ophtha and DIARETDB1 datasets which are publicly available. Also, the accuracy of the red-lesion detection methods is mostly evaluated with sensitivity and specificity parameters. The sensitivity of a method is determined by its capability to correctly identify lesions and specificity measures the degree of correctly identifying non-lesions. Supplementary Table S1 summarizes the datasets and experimental outcomes of the related existing methods.

The main contributions of our proposed method are summarized in several points.

- In this paper, all the boundary pixels are extracted using simple pixel-wise computations. Then, these boundary pixels are utilized in the red-lesion detection process.
- It is possible to control the number of pixels considered as the boundary ones to prevent from producing superfluous pixels. As the number of extracted boundary pixels increases, the required processing time for detecting red-lesion increases. However, if the number of extracted boundary pixels is low, the accuracy of detecting red-lesions is reduced and some red-lesions are missed. Therefore, the number of extracted boundary pixels should be equal to a value which makes a trade-off between processing time and detection accuracy.
- Red-lesions are considered as intensity changes in all directions. In fact, an important distinguishing feature of blood vessels is that there is at least one direction in the boundaries of blood vessels along which no significant intensity change is observed. This is while in red-lesions it is possible to observe intensity change along almost all directions.
- Therefore, the neighbour windows around the boundary pixels of red-lesions have different intensity-based features.
- In this paper, a novel optic disc localization algorithm is proposed to identify the location of optic disc with simple computations. The importance of proposing this algorithm is to remove superfluous boundary pixels which are mistakenly considered as the boundary pixels for red-lesions. With respect to the optic disc zone, it should be noted that no red-lesion is usually observed inside this zone. Therefore, if the optic disc zone is correctly localized, any boundary pixel which is located inside it is removed and the production of false positive cases inside the optic disc zone is prevented.

The experimental results show that the proposed method has an improved performance in terms of speed and accuracy in comparison with the related state-of-the-art research works.

## Method

In this section, the proposed method for red-lesion extraction from fundus images is explained in details. The study was approved by the Ethics Committee of National Institute for Medical Research Development (IR.NIMAD.REC.1398.064).

**Figure 1:**
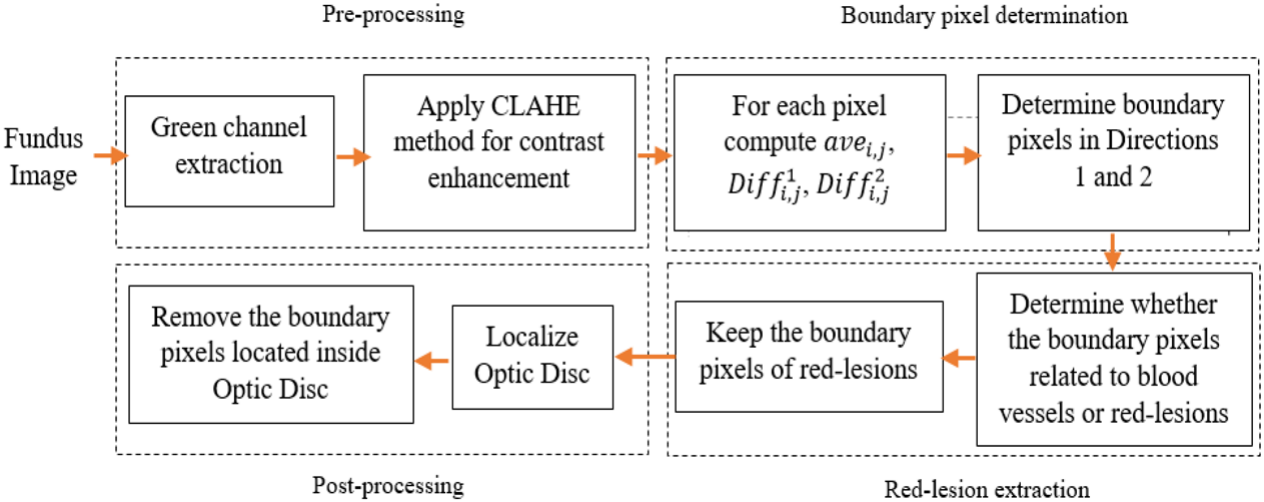
Block diagram for REDICA method.

The novel method called Red-lesion Extraction with Directional Intensity Changes’ Analysis (REDICA) consists of four main phases. The main phases are pre-processing, boundary pixel determination, red-lesion extraction, and post-processing. Figure 1 presents the block diagram for REDICA method. All phases are explained in the following.

### Pre-processing

In this phase, the quality of fundus image is improved and the image is prepared for processing in the next phases. The method used here for contrast enhancement is Contrast Limited Adaptive Histogram Equalization (CLAHE). It should be mentioned that CLAHE originates from Adaptive Histogram Equalization (AHE) which in turn is a kind of histogram equalization. It should be mentioned that AHE utilizes several histograms each of them is related to a distinct region of image to improve the contrast of image. Using AHE method, the local contrast of image is improved and the edges in each region of image are enhanced. However, the disadvantage of AHE method is to over-amplify the noise in the process of contrast enhancement. This issue is solved in CLAHE method by the limited amplification of contrast. This method has been utilized in a large number of research works with similar purposes^45–48^. At first, the green channel of the fundus image is extracted and then CLAHE method is applied on it. The reason for extracting green channel is that among red, blue and green channels of image, green channel has the best contrast. Since the proposed method works based on the analysis of intensity, the provision of improved contrast for image is very important and significantly helps in the process of red-lesion extraction. Between red and green channels, green channel has a significantly better contrast and the improved contrast of image leads to the provision of more boundary pixels in the boundary pixel determination phase of the proposed method. This in turn leads to the extraction of red-lesions with higher accuracy. In addition, with respect to the blue channel, it should be mentioned that the blue channel of fundus image is very noisy and it is necessary to reduce the noise level before processing the image for the purpose of red-lesion detection. This noise reduction should be performed through some de-noising method which in turn inflicts an extra computational load.

### Boundary pixel determination

In this phase, all the boundary pixels in the fundus images are extracted. These pixels include the boundary pixels for blood vessels and red lesions.

In order to explain the method proposed for this phase, several notations should be defined. Let *I* denote a fundus image with *m* rows and *n* columns. The pixel located at (*i*,*j*) is denoted with ${p}_{i,j}$. The intensity value of ${p}_{i,j}$ is denoted by ${X}_{i,j}$. The boundary pixels are the ones where a significant transition occurs in the intensity values of image pixels. Therefore, it sounds reasonable to evaluate the intensity changes in several different directions and pick the highest intensity changes.

In order to determine the highest intensity changes, we define a neighborhood window for each pixel. Let ${N}_{i,j}$ denote the neighborhood window for ${p}_{i,j}$. This window is considered as a ${s}_{w}$ × ${s}_{w}$ diamond centered at ${p}_{i,j}$. The average intensity value of neighbors of ${p}_{i,j}$ is denoted with ${ave}_{i,j}$. It can be computed via the following equation.

1 $${ave}_{i,j}=\frac{\sum_{\forall {p}_{i,j}\epsilon {N}_{i,j}}{X}_{i,j}}{\left(\frac{{{s}_{w}}^{2}+1}{2}\right)}.$$

In order to verify the directional intensity changes of the image, we consider two directions the first of which is 135 degrees and is called Direction 1. Also, the second one is 45 degrees and is called Direction 2. In order to verify image in Direction 1, for each pixel we compute the difference between the average intensity of two windows consecutively located at the north-west and south-east sides of the pixel. Let ${Diff}_{i,j}^{1}$ denote the absolute difference between the average values of two near windows around ${p}_{i,j}$ in Direction 1. In fact, the following equation is true.

2 $${Diff}_{i,j}^{1}=\left|{ave}_{i-1,j-1}-{ave}_{i+1,j+1}\right|.$$

It should be mentioned that the pixels which maximize the value of ${Diff}_{i,j}^{1}$ are the boundary pixels in Direction 1. In fact, ${p}_{i,j}$ is considered to be a boundary pixel in Direction 1, if ${Diff}_{i,j}^{1}>{Diff}_{i-1,j-1}^{1}$ and ${Diff}_{i,j}^{1}>{Diff}_{i+1,j+1}^{1}$ are true. The set of all boundary pixels locally maximizing ${Diff}_{i,j}^{1}$ are denoted with *P*_1_.

In addition, for each pixel we compute the difference between average intensity of two windows consecutively located at the north-east and south-west sides of Direction 2. Let ${Diff}_{i,j}^{2}$ denote the absolute difference between the average values of two near windows around ${p}_{i,j}$ in Direction 2. In fact, the following equation is true.

3 $${Diff}_{i,j}^{2}=\left|{ave}_{i-1,j+1}-{ave}_{i+1,j-1}\right|.$$

It is worth pointing out that the pixels which maximize the value of ${Diff}_{i,j}^{2}$ are the boundary pixels in Direction 2. In fact, ${p}_{i,j}$ is considered to be a boundary pixel in Direction 2, if ${Diff}_{i,j}^{2}>{Diff}_{i-1,j+1}^{2}$ and ${Diff}_{i,j}^{2}>{Diff}_{i+1,j-1}^{2}$ are true. The set of all boundary pixels locally maximizing ${Diff}_{i,j}^{2}$ are denoted with *P*_2_. It is necessary to compute the values of ${Diff}_{i,j}^{1}$ and ${Diff}_{i,j}^{2}$ for all ${p}_{i,j}$ s to determine whether or not they are boundary pixels for Directions 1 and 2, respectively. The pseudo-code for boundary pixel determination phase is shown in Algorithm 1. In order to restrict the number of boundary pixels and remove extra points corresponding to negligible intensity transitions in the fundus image, we consider a threshold value denoted by ${th}_{Diff}$. Then, only the boundary pixels for which the values of ${Diff}_{i,j}^{1}$ or ${Diff}_{i,j}^{2}$ are higher than ${th}_{Diff}$, are selected as the boundary ones for Direction 1 or Direction 2, respectively. For simplicity, the conditions of ${Diff}_{i,j}^{1}>{Diff}_{i-1,j-1}^{1}$, ${Diff}_{i,j}^{1}>{Diff}_{i+1,j+1}^{1}$ and ${Diff}_{i,j}^{1}>{th}_{Diff}$ are denoted with *R*_1_, *S*_1_ and *T*_1_, respectively. Also, the conditions of ${Diff}_{i,j}^{2}>{Diff}_{i-1,j+1}^{2}$, ${Diff}_{i,j}^{2}>{Diff}_{i+1,j-1}^{2}$ and ${Diff}_{i,j}^{2}>{th}_{Diff}$ are denoted with *R*_2_, *S*_2_ and *T*_2_, respectively.
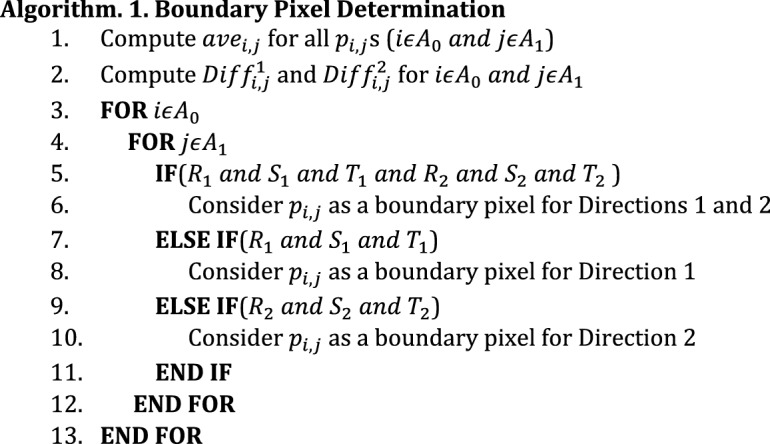


At first, the values of ${ave}_{i,j}$ are computed for all ${p}_{i,j}$ s (line 1 of Algorithm 1). It should be noted that since a neighborhood window must be considered around pixel, it is not possible to compute ${ave}_{i,j}$ for marginal pixels. In fact, the value of *i* lies in the range of $\left[ {\left( {\left[ {\frac{{s_{w} }}{2}} \right] + 1} \right),\,\left( {m - \left[ {\frac{{s_{w} }}{2}} \right]} \right)} \right]$. Also, the value of *j* should be in the range of $\left[ {\left( {\left[ {\frac{{s_{w} }}{2}} \right] + 1} \right),\,\left( {n - \left[ {\frac{{s_{w} }}{2}} \right]} \right)} \right]$. For simplicity, $\left[ {\left( {\left[ {\frac{{s_{w} }}{2}} \right] + 1} \right),\,\left( {m - \left[ {\frac{{s_{w} }}{2}} \right]} \right)} \right]$ and $\left[ {\left( {\left[ {\frac{{s_{w} }}{2}} \right] + 1} \right),\,\left( {n - \left[ {\frac{{s_{w} }}{2}} \right]} \right)} \right]$ intervals are denoted with *A*_*0*_ and *A*_*1*_, respectively. Then, the values of ${Diff}_{i,j}^{1}$ and ${Diff}_{i,j}^{2}$ are computed for ${p}_{i,j}$ s (line 2 of Algorithm 1). A loop is written in which all pixels are verified to determine whether or not they can be considered as boundary pixels for Direction 1 and Direction 2 (lines 3 to 13 of Algorithm 1). If all the conditions of *R*_1_, *S*_1_, *T*_1_, *R*_2_, *S*_2_ and *T*_2_ are true, ${p}_{i,j}$ can be considered as a boundary pixel for both directions (lines 5 and 6). If only the conditions of *R*_1_, *S*_*1*_ and *T*_1_ are true, ${p}_{i,j}$ can be considered as a boundary pixel for Direction 1 (lines 7 and 8). Also, if only the conditions of *R*_*2*_, *S*_*2*_ and *T*_*2*_ are true, ${p}_{i,j}$ can be considered as a boundary pixel for Direction 2 (lines 9 and 10).

### Red-lesion extraction

In this section, the unique features of the boundary pixels of red lesions are extracted from the fundus retinal image and are utilized for discriminating them from other boundary pixels.

In the previous section, all the boundary pixels were found through the pseudo-code of Algorithm 1. However, it is necessary to look for the distinguishing properties of red-lesion boundaries to discriminate them from other boundary pixels specially the boundary pixels of blood vessels.

**Figure 2:**
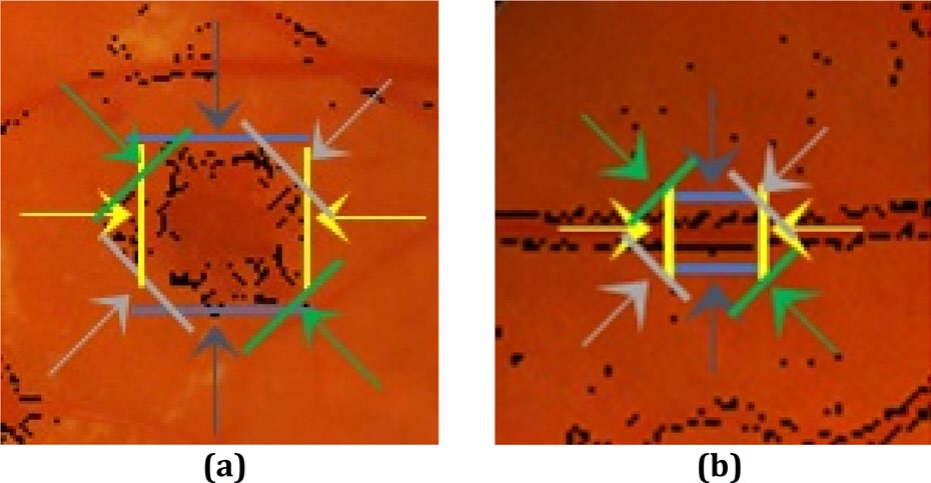
Directional intensity changes in (**a**) red-lesion, (**b**) blood vessel.

With the accurate analysis of red-lesions in fundus retinal images, it is observed that red-lesions can be seen as intensity changes in almost all directions. However, for blood vessels, there is at least one direction that no intensity change is observed along that direction. Figure 2 presents this point which is considered as the most important distinguishing feature between boundary pixels. In this figure, intensity changes in different directions are shown with different colors. In part a, a red-lesion is presented and it can be observed that in different directions, there are significant intensity changes. However, in part b, a blood vessel is shown and it can be seen that in one direction (yellow direction) no significant change is observed.

This feature can be used in the discrimination between blood vessel boundary pixels and red-lesion boundary pixels. In continue, we explain how this feature is utilized in red-lesion extraction.

Let *P* denote the set of all boundary pixels obtained in the previous phase using the pseudo-code of Algorithm. 1. All pixels which belong to *P* should be verified to determine whether they are boundary ones for blood vessels or red lesions. For each ${p}_{i,j}\epsilon P$, several directions are considered and along each direction several neighborhood window are considered to measure the intensity changes in the direction. Let ${G}_{{i}_{0}:{i}_{1}}^{{j}_{0}:{j}_{1}}=$ g(${i}_{0}:{i}_{1},{j}_{0}:{j}_{1}),1\le {i}_{0},{i}_{1}\le m,1\le {j}_{0},{j}_{1}\le n$ denote a part of image with rectangle shape which includes rows from ${i}_{0}$ to ${i}_{1}$ and columns from ${j}_{0}$ to ${j}_{1}$. At first, consider Direction 0 which makes 0-degree angle with x axis. Let ${W}_{i,j}^{k,0} (k=0,\pm 1,\pm 2,\dots ,\pm num)$ denote the *k*th neighborhood window of ${p}_{i,j}$ in Direction 0. Let *d*_0_ denote the width of neighborhood window. The neighborhood windows are considered to be square. Also, *num* denotes the number of neighborhood windows considered at one side of ${p}_{i,j}$. ${W}_{i,j}^{k,0}$ is determined using the following equation.

4 $${W}_{i,j}^{k,0}=g\left({x}_{0}:\left({x}_{0}+{d}_{0}\right),\left(j+kr\right):\left(j+kr+{d}_{0}\right)\right),$$

where in (4) ${x}_{0}$ denotes the number of first row in ${W}_{i,j}^{\text{0,0}}$. Also, *r* is a parameter which determines the overlap between adjacent windows. Note that ${W}_{i,j}^{\text{0,0}}$ is determined according to the following rule.

5 $${W}_{i,j}^{\text{0,0}}=\left\{\begin{array}{l}{G}_{i-{d}_{0}:i}^{j-{d}_{0}:j}, if\, mean\left({G}_{i-{d}_{0}:i}^{j-{d}_{0}:j}\right)=min\_all\\ {G}_{i-{d}_{0}:i}^{j-(\frac{{d}_{0}}{2}):j+(\frac{{d}_{0}}{2})}, if \,mean\left({G}_{i-{d}_{0}:i}^{j-(\frac{{d}_{0}}{2}):j+(\frac{{d}_{0}}{2})}\right)=min\_all\\ {G}_{i-{d}_{0}:i}^{j:j+{d}_{0}}, if \,mean\left({G}_{i-{d}_{0}:i}^{j:j+{d}_{0}}\right)=min\_all\\ {G}_{i:i+{d}_{0}}^{j-{d}_{0}:j}, if\, mean\left({G}_{i:i+{d}_{0}}^{j-{d}_{0}:j}\right)=min\_all\\ {G}_{i:i+{d}_{0}}^{j-(\frac{{d}_{0}}{2}):j+(\frac{{d}_{0}}{2})}, if\, mean\left({G}_{i:i+{d}_{0}}^{j-(\frac{{d}_{0}}{2}):j+(\frac{{d}_{0}}{2})}\right)=min\_all \\ {G}_{i:i+{d}_{0}}^{j:j+{d}_{0}}, if \,mean\left({G}_{i:i+{d}_{0}}^{j:j+{d}_{0}}\right)=min\_all \end{array}.\right.$$

In (5) $mean$ is the averaging operator. For instance, $mean\left({G}_{i:i+{d}_{0}}^{j:j+{d}_{0}}\right)$ denotes the average of intensity levels of pixels (in g channel) which belong to ${G}_{i:i+{d}_{0}}^{j:j+{d}_{0}}$. Also, in (5) $min\_all$ denotes the minimum value of $mean\left({G}_{i-{d}_{0}:i}^{j-{d}_{0}:j}\right)$, $mean\left({G}_{i-{d}_{0}:i}^{j-(\frac{{d}_{0}}{2}):j+(\frac{{d}_{0}}{2})}\right)$, $mean\left({G}_{i-{d}_{0}:i}^{j:j+{d}_{0}}\right)$, $mean\left({G}_{i:i+{d}_{0}}^{j-{d}_{0}:j}\right)$, $mean\left({G}_{i:i+{d}_{0}}^{j-(\frac{{d}_{0}}{2}):j+(\frac{{d}_{0}}{2})}\right)$ and $mean\left({G}_{i:i+{d}_{0}}^{j:j+{d}_{0}}\right)$.

After describing neighborhood windows for Direction 0, we consider Direction θ which makes θ degrees angle with x axis. Let ${W}_{i,j}^{k,\theta} (k=0,\pm 1,\pm 2,\dots )$ denote the *k*th neighborhood window of ${p}_{i,j}$ in Direction θ. Note that ${W}_{i,j}^{0,\theta}$ is equal to ${W}_{i,j}^{\text{0,0}}$ in (4).

Let ${x}_{\theta}$ and ${y}_{\theta}$ denote the number of first row and the number of first column in ${W}_{i,j}^{0,\theta}$, respectively. ${W}_{i,j}^{k,\theta}$ can be obtained through the following equation.

6 $${W}_{i,j}^{k,\theta}\left(0<{\uptheta}<90\right)=g\left({X}_{\theta}:\left({X}_{\theta}+{d}_{0}\right),{Y}_{\theta }^{+}:\left({Y}_{\theta }^{+}+{d}_{0}\right)\right),$$

7 $${W}_{i,j}^{k,\theta}\left(90<{\uptheta}<180\right)=g\left({X}_{\theta}:\left({X}_{\theta}+{d}_{0}\right),{Y}_{\theta }^{-}:\left({Y}_{\theta }^{-}+{d}_{0}\right)\right),$$

where in (6) and (7) ${X}_{\theta}={x}_{\theta}-kr$, ${Y}_{\theta }^{+}={y}_{\theta}+\left[kr\,{\tan}\theta\right]$ and ${Y}_{\theta }^{-}={y}_{\theta}-\left[kr\,{\tan}\theta\right]$ are used for summarizing the equations.
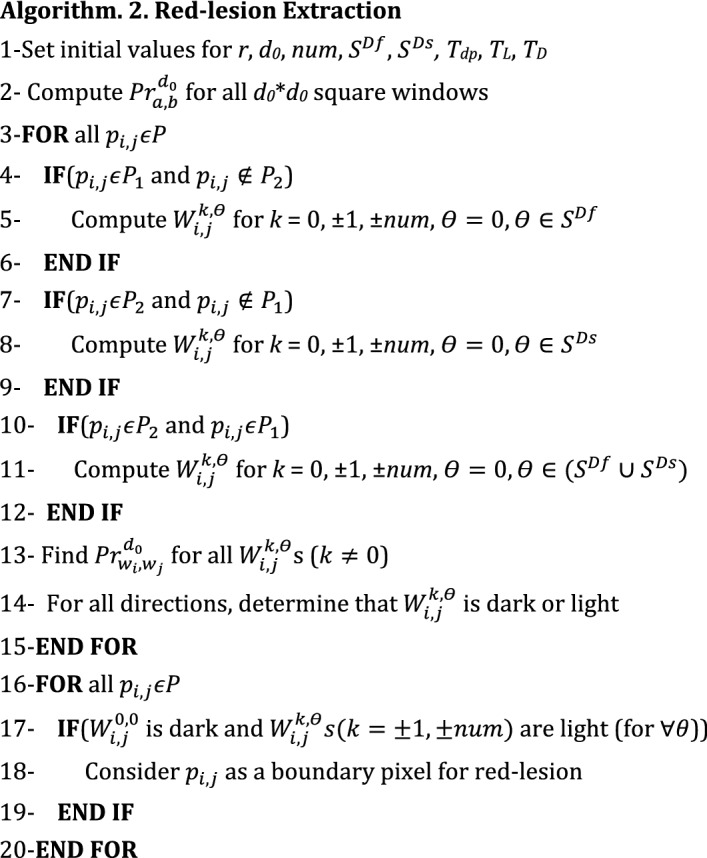


The pseudo-code for red-lesion extraction phase is presented in Algorithm 2. It should be mentioned that it is not necessary to verify all directions for each boundary pixel. In fact, it is sufficient to verify several directions in the first quarter ($0<{\uptheta}<90$) or in the second quarter (90 $<{\uptheta}<180$) for each boundary pixel. The reason is that in Section III. B the boundary pixels in Direction 1 (135 degrees) and Direction 2 (45 degrees) have been determined. In other words, for a boundary pixel *p*_*i,j*_ maximizing ${Diff}_{i,j}^{1}$, it is sufficient to verify several directions in the first quarter. Also, for a boundary pixel *p*_*i,j*_ maximizing ${Diff}_{i,j}^{2}$, it is sufficient to verify several directions in the second quarter. Let ${S}^{Df}$ and ${S}^{Ds}$ denote the set of all angles considered for verifying different directions in the first and second quarter, respectively. The mean intensity value of ${W}_{i,j}^{k,\theta}$ is denoted by ${m}_{i,j}^{k,\theta}$. At first, all the required parameters such as *r*, *d*_0_, *num*, ${S}^{Df}$ and ${S}^{Ds}$ are initialized. Then, it is necessary to verify all *d*_0_ × *d*_0_ squares in the image from darkness or lightness point of view. For each *d*_0_ × *d*_0_ square, a new parameter is calculated which characterizes the percentage of dark pixels. By “dark pixel”, we mean a pixel the intensity of which is lower than a pre-defined threshold. Let us denote such a pre-defined threshold for defining a dark pixel with *T*_*dp*_. A square window is considered as a dark one if the percentage of its dark pixels is higher than a pre-defined threshold denoted by *T*_*D*_. Also, a square window is considered as a light one if the percentage of dark pixels is lower than another pre-defined threshold denoted by *T*_*L*_. Let ${Pr}_{a,b}^{{d}_{0}}$ denote the percentage of dark pixels in a *d*_0_ × *d*_0_ square window starting from row *a* and column *b*. In line 2, we compute ${Pr}_{a,b}^{{d}_{0}}$ for all *d*_0_ × *d*_*0*_ square windows and determine whether the related square window is dark or light after comparing ${Pr}_{a,b}^{{d}_{0}}$ with *T*_*L*_ and *T*_*D*_. In line 1, *T*_*dp*_, *T*_*L*_ and *T*_*D*_ are also initialized.

Then, a loop is executed in which for every pixel ${p}_{i,j}\epsilon P$ different neighborhood windows (${W}_{i,j}^{k,\theta}$ s) are built (lines 3 to 15 of Algorithm 2) depending on whether the pixel belongs to *P*_1_ or *P*_2_. It should be reminded that the set of all boundary pixels locally maximizing ${Diff}_{i,j}^{1}$ and ${Diff}_{i,j}^{2}$ are denoted with *P*_1_ and *P*_2_, respectively. Also, is the set of all boundary pixels obtained in the previous phase using the pseudo-code of Algorithm. 1. If the pixel belongs to *P*_1_ (*P*_2_) and does not belong to *P*_2_ (*P*_1_), all the windows (${W}_{i,j}^{k,\theta}$ s) in the directions of ${S}^{Df} ({S}^{Ds})$ should be verified (lines 4 to 9 in Algorithm 2). If the pixel belongs to both *P*_1_ and *P*_2_, all the windows (${W}_{i,j}^{k,\theta}$ s) in the directions of ${S}^{Df}$ or ${S}^{Ds}$ should be verified (lines 10 to 12 in Algorithm 2). In all cases, it is necessary to verify the windows in Direction 0 (${W}_{i,j}^{k,0}$). In line 13, for each ${W}_{i,j}^{k,\theta}$, the values of ${Pr}_{{w}_{i},{w}_{j}}^{{d}_{0}}$ is found using line 2 and it is determined whether the related ${W}_{i,j}^{k,\theta}$ is dark or light (line 14). If for all directions, ${W}_{i,j}^{\text{0,0}}$ is dark and other neighbor windows including ${W}_{i,j}^{k,\theta}$ s (*k* $\ne$ 0) are light, ${p}_{i,j}$ is considered as a boundary pixel for a red-lesion (lines 17 to 19).

### Post-processing

In this part, it is necessary to remove pixels which are mistakenly considered as the boundary pixel for red lesions in the previous phase. Here, we suggest a new algorithm for localizing the optic disc in the fundus retinal image. If we can localize the optic disc zone, we can remove the boundary pixels which are located inside this zone. The reason is that there is usually no red-lesion inside the optic disc. The main point utilized in the optic disc localization is that this zone contains the lightest part of image and also the aggregation of blood vessels which are comparatively dark. Therefore, if the standard deviation value for the intensity values of the pixels inside optic disc is computed, it has a high value for this zone. It is possible to grid image and perform computations separately for each grid to determine the grid with the highest variance value. Let *d*_*od*_ denote the approximate diameter of optic disc. This parameter can be computed from several fundus images. Our purpose is to find a ${d}_{od} \times {d}_{od}$ square approximately located on the optic disc zone. The pseudo-code for optic disc localization is presented in Algorithm 3.
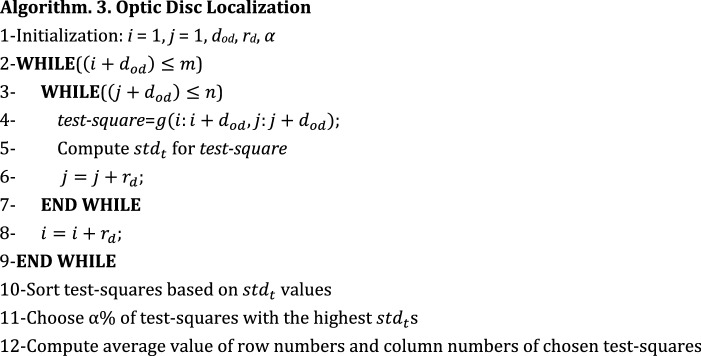


At first, the required parameters are set. Note that *r*_*d*_ denotes the distance between two adjacent ${d}_{od}\times {d}_{od}$ squares. In fact, *r*_*d*_ is equal to the number of rows (columns) between the starting rows (columns) of two adjacent squares. If *r*_*d*_ is equal to 1, all the possible squares with ${d}_{od}\times {d}_{od}$ dimensions in image are considered and tested. However, it may result in huge volume of computations. Therefore, it sounds reasonable to choose a higher value for *r*_*d*_. It is obvious that the number of overlapped rows and columns between two adjacent squares is equal to $({d}_{od}-{r}_{d})$.

Then, a loop is executed in which all test-squares are built (lines 2 to 9 in Algorithm 3). By “test-square”, we mean a ${d}_{od}\times {d}_{od}$ square tested for being the optic disc zone. Also, for each test-square the value of standard deviation of intensity values of its pixels is computed (line 5 in Algorithm 3). Let *std*_*t*_ denotes the standard deviation of intensity values of a test-square. ${std}_{t}$ is equal to $\sqrt{{\sum }_{{p}_{i,j}\epsilon test-square}{\left({X}_{i,j}-\overline{X }\right)}^{2}/({{d}_{od}}^{2}-1)}$ where $\overline{X }$ is the average intensity value of all ${p}_{i,j}$ s which belong to the test-square. The test-squares are sorted in a descending order based on the values of ${std}_{t}$ (line 10 in Algorithm 3). Then, *α* percent of test-squares with the highest values of ${std}_{t}$ are chosen (line 11 in Algorithm 3). The starting row numbers and the starting column numbers of the selected test-squares are averaged to determine the final test-square (line 12 in Algorithm 3). The final test-square is considered as the optic disc zone.

## Numerical results

In this section, the performance of REDICA method is evaluated. In order to evaluate the performance of the proposed method, a program has been run in MATLAB. Also, the code has been tested for three different datasets including Diaretdb1^49^, Diaretdb0^50^ and Kaggle datasets^51^. Table 1 presents the initial values for the required parameters.

**Table 1:** The initial values for the required parameters.

Parameter	Value
${d}_{od}$	180
${th}_{Diff}$	0.12
${s}_{w}$	5
${d}_{0}$	10
*m*	1152
*n*	1500
*num*	1
*T*_*dp*_	0.2
*T*_*D*_	0.7
*T*_*L*_	0.4
${r}_{d}$	10

In addition, it should be mentioned that the distribution used in CLAHE method is uniform and the clip limit is set to 0.02.

**Figure 3:**
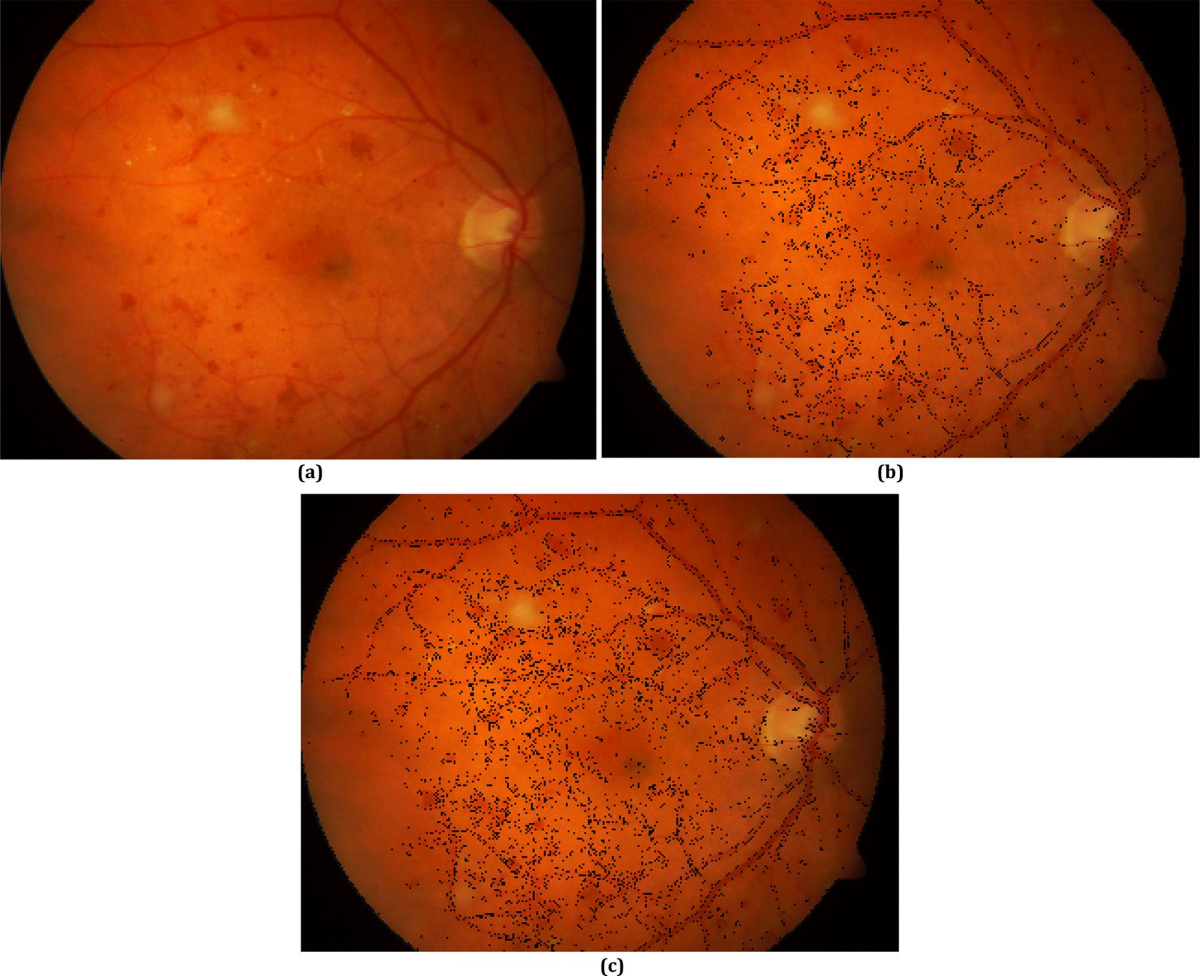
(**a**) a fundus image, boundary pixels for (**b**) *th*_*Diff*_ = 0.12, (**c**) *th*_*Diff*_ = 0.08.

Figure 3 presents a fundus image from Diaretdb1 dataset versus its boundary pixels which are obtained through boundary pixel determination phase in Algorithm. 1. In parts (b) and (c), the boundary pixels are obtained for ${th}_{Diff}$ = 0.12 and ${th}_{Diff}$ = 0.08, respectively. It is obvious that the more the value of ${th}_{Diff}$, the less the number of obtained boundary pixels. Thus, ${th}_{Diff}$ should be set to a value that provide sufficient number of boundary pixels. The reason is that if the number of boundary pixels is considerably high, the processing time for red-lesion extraction becomes high while it does not improve the accuracy significantly.

**Figure 4:**
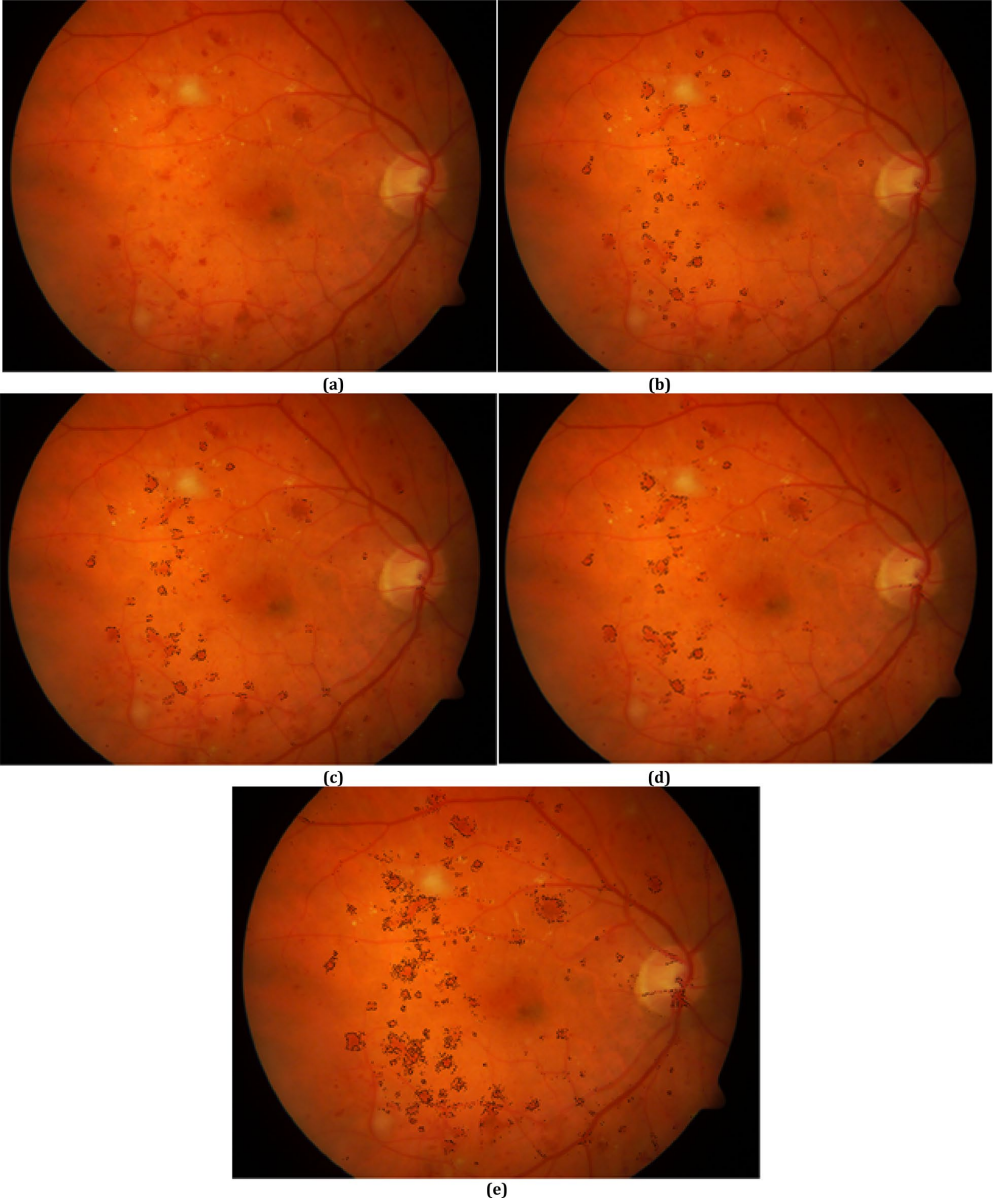
(**a**) A fundus image, boundary pixels for red-lesions for (**b**) *d*_0_ = 10, (**c**) *d*_0_ = 16, (**d**) *d*_0_ = 22, (**e**) all boundary pixels for red-lesions.

Figure 4 presents a fundus image and its boundary pixels of red-lesions obtained from the red-lesion extraction phase of REDICA method. As can be seen, the boundary pixels in parts *b*, *c*, *d* are obtained for *d*_0_ = 10, 16, 22, respectively. Also, part *e* presents all the boundary pixels for red lesions. As obvious from the figure, the boundary pixels for red lesions are well extracted from visual point of view.

**Figure 5:**
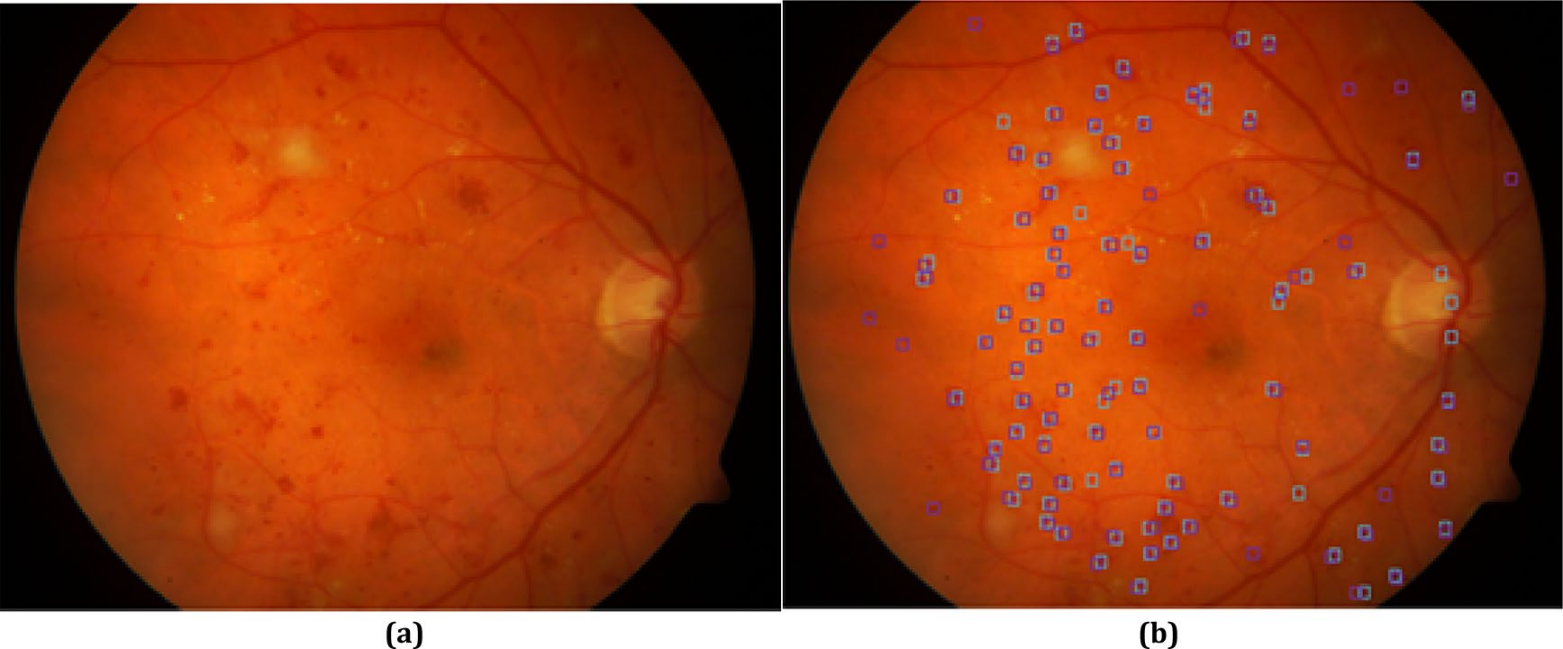
(**a**) A fundus retinal image, (**b**) localized red lesions by REDICA algorithm presented with blue squares and by ophthalmologist by purple squares.

Figure 5 presents a fundus retinal image versus the locations of red-lesions localized by the proposed algorithm. The localized red-lesions extracted by REDICA algorithm are shown with blue squares. Also, the red-lesions localized by the ophthalmologists are shown with purple squares. It can be seen that red-lesions in the REDICA algorithm are extracted with good accuracy.

**Figure 6:**
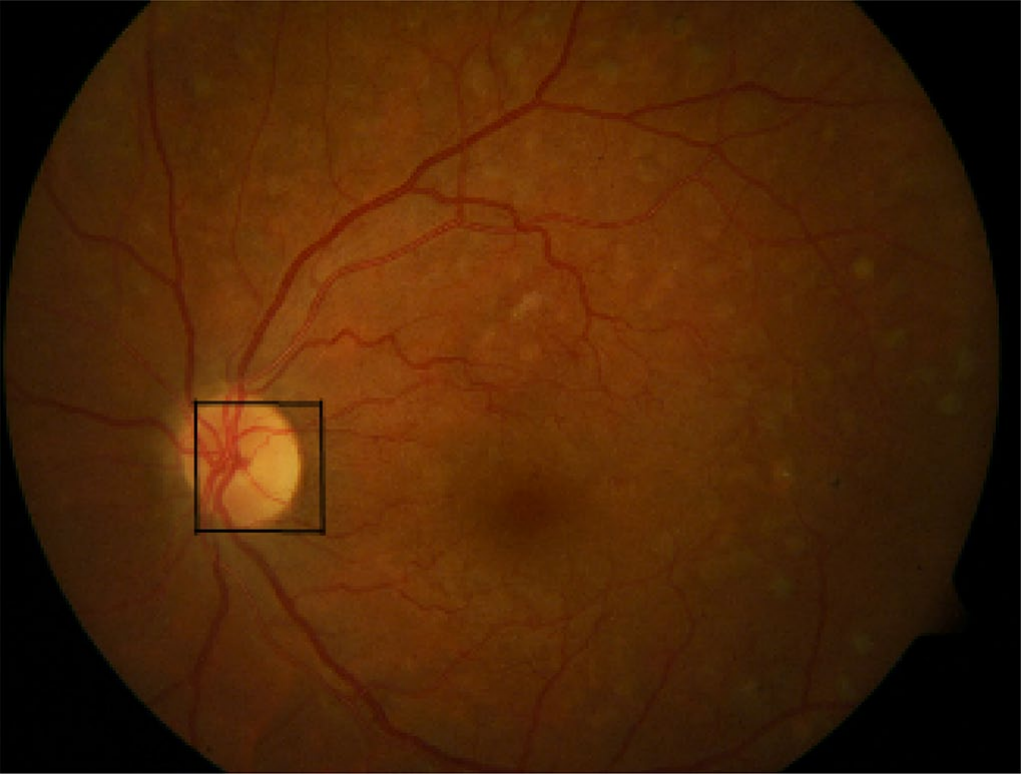
A fundus image and its localized optic disc.

Figure 6 presents a fundus retinal image for which the optic disc zone is localized using the pseudo-code of Algorithm 3. The optic disc zone is shown with a black square in the image.

With respect to the quantitative analysis of the proposed method, we have computed the values of specificity (*SP*) and sensitivity (*SE*) which are two important metrics^52^. Specificity and sensitivity are defined through the following equations.

8 $$SP=\frac{TN}{TN+FP},$$

9 $$SE=\frac{TP}{TP+FN},$$

where in (8) and (9), *TP*, *TN*, *FP* and *FN* denote truly classified lesions, correctly classified non-lesions, non-lesions falsely classified as lesions, and lesions falsely classified as non-lesions, respectively.

In order to analyze the statistical difference between the results of our proposed method and the existing methods, a threshold is considered for *p*-value which is equal to 0.05. It should be mentioned that in the datasets used here, the red-lesions are localized and not exactly segmented. Therefore, in order to determine the values of *TP*, *TN*, *FP* and *FN*, after extracting the boundary pixels related to red-lesions, it is verified whether or not they are included inside a red-lesion localized and annotated by the ophthalmologist in the dataset. If the mentioned boundary pixel is located inside the spot, such spot is considered as a correctly detected red-lesion by the proposed algorithm (*TP*). If for a red-lesion annotated by the ophthalmologist, no pixel has been extracted by the proposed algorithm, the mentioned spot is considered as a false negative (*FN*). In addition, if a boundary pixel is not located inside any annotated red-lesion, it is considered as a false positive spot (*FP*). Furthermore, if a pixel which is not determined as a boundary one for red-lesion is located inside a non-annotated regions of fundus image, it is considered as a true negative (*TN*). It should be also mentioned that the accuracy parameters including specificity and sensitivity are computed based on the number of lesions.

In continue, we notice to the results related to Diaretdb1 dataset. Diaretdb1 consists of 89 images that 5 images are normal and the others have the sign of DR. The research works chosen for comparison for Diaretdb1 dataset are the research works of Refs.^35,53^. Dark and bright abnormal lesions are detected with the method of Ref.^53^. In the method of Ref.^53^, the whole fundus image is divided into patches and for each patch a series of features are extracted based on texture and morphology of the patch. In order to extract texture feature, rotation-invariant Local Binary Pattern (LBP) is utilized which is a type of LBP texture indicators. The features related to the morphology are extracted based on granulometry technique. Then, several different classifiers including Random Forests (RF), Gaussian processes and SVM utilizes the extracted features to categorize the abnormal pathologies. The capability of method was evaluated on DIARETDB1 in term of detecting hemorrhages and micro-aneurysms. In Table 2 the results of comparison between REDICA and the methods of Refs.^35,53^ are presented. It was shown that the sensitivity and specificity values in the method of Ref.^53^ are equal to 0.75 and 0.75, respectively which are significantly lower than those of our proposed algorithm.

**Table 2:** *SE* and *SP* values of the methods of Ref.^35,53^ for Diaretdb1, the methods of Ref.^33,54^, for Diaretdb0, the method^43^ for Kaggle, and the REDICA method for all datasets.

Dataset\method	REDICA	Method of Ref.^53^	Method of Ref.^35^	Method of Ref.^54^	Method of Ref.^33^	Method of Ref.^43^
Diaretdb1	*SE* = 0.87 *SP* = 0.88	*SE* = 0.75 *SP* = 0.75	*SE* = 0.88 *SP* = 0.91			
Diaretdb0	*SE* = 0.89 *SP* = 0.9			*SE* = 0.95 *SP* = 0.82	*SE* = 0.74 *SP* = 0.85	
Kaggle	*SE* = 0.82 *SP* = 0.9					*SE* = 0.83 *SP* = 0.85

In addition, the method of Ref.^35^ evaluated its performance on DIARETDB1. The sensitivity and specificity values of method of Ref.^35^ are equal to 0.88 and 0.91, respectively which are very close to the values of same parameters in our proposed algorithm. It should be mentioned that the required computations in our proposed method are considerably lower compared to the method of Ref.^35^. The reason is that the method of Ref.^35^ requires the segmentation of vessels and the decomposition of image into several layers before determining lesions which in turn affects execution speed and time.

The research work chosen for comparison for Kaggle dataset is Ref.^43^. In Ref.^43^ a CNN is trained with a number of informative samples from a large number of medical images. A dynamic weight is assigned to each pixel to present its informativeness level. After each round of CNN training, the weight of each pixel is updated. The final CNN is capable to categorize each pixel in a test image. It should be mentioned that the number of images used for training and testing stages in SeS method is equal to 5287 and 1392, respectively. The number of images randomly selected from the same dataset in REDICA is considered to be 1392, too. The values of sensitivity and specificity for REDICA method and SeS method of Ref.^43^ are presented in Table 2. As can be observed, specificity and sensitivity values in REDICA method are 0.9 and 0.82, respectively. Also, specificity and sensitivity values in SeS method are equal to 0.85 and 0.83, respectively. It can be seen in Table 2 that the sensitivity values of both methods are very close and the specificity value of REDICA is higher than the SeS method of Ref.^43^.

In the following, we pay attention to the results related to Diaretdb0 dataset. Diaretdb0 includes 130 images 20 of them are normal and 110 of them have the signs of DR. The research works which are selected for comparison for Diaretdb0 dataset are Refs.^33,54^. In Ref.^54^ a method is proposed for extracting hemorrhages from retinal fundus images. At first, all dark regions including blood vessels and hemorrhages are extracted from the image. Then, retinal vessels are segmented and gradually removed from the extracted regions. Table 2 presents the values of sensitivity and specificity for REDICA, the method of Ref.^54^ and the method of Ref.^33^ for Diaretdb0 dataset. It can be observed that the values of sensitivity and specificity for the method of Ref.^54^ are equal to 0.95 and 0.82, respectively. Also, REDICA is capable of providing the values of 0.89 and 0.9 for sensitivity and specificity, respectively. The value of specificity in our method is higher than the method of Ref.^54^. With respect to sensitivity value, it should be noted that the method of Ref.^54^ requires the exact segmentation of blood vessels which in turn inflicts a large volume of computations while there is no need to the segmentation in REDICA. In Ref.^33^ a method for detecting different kinds of abnormalities in retina including red-lesions is proposed. The method called HEM detector, works based on the analysis of several wavelet bands and Hessian multi-scale analysis. Large structures like red-lesions and blood vessels can be detected in high wavelet levels. Also, with the help of Hessian multi-scale analysis, it is possible to find blob-like structures in the image. It can be observed that the values of sensitivity and specificity for REDICA method are better than those of the method of Ref.^33^.

Also, the Receiver Operating Characteristic (ROC) curves of the proposed algorithm are presented for all datasets. These curves can be observed in Supplementary Fig. S1. In order to draw the ROC curves, we have changed the value of *T*_*D*_ and verified its effect on the specificity and sensitivity values. The increment of *T*_*D*_ makes more difficult conditions for considering a boundary pixel as a boundary pixel for a red-lesion. The reason is that the related patch located in the center should be darker in this case. Therefore, the number of boundary pixels extracted as the boundary pixels for red-lesions is reduced and the SE value also decreases. In such conditions, the number of boundary pixels falsely detected as the boundary pixels for red-lesions also decreases. Thus, the specificity value increases. The ROC curves present such a relationship between the values of *SE* and (1 − *SP*). In addition, the Area Under ROC Curve (AUC) is computed for REDICA method for different datasets. It can be observed that AUC values for Diaretdb0, Diaretdb1 and Kaggle datasets are equal to 0.89, 0.87 and 0.9, respectively. In the methods of Refs.^43,53^, AUC values for Diaretdb1 and Kaggle datasets are equal to 0.83 and 0.89, respectively.

## Conclusion

In this paper, a novel method for red-lesion extraction from fundus retinal images is proposed. The method works based on the analysis of intensity changes in the image. Firstly, the method improves the contrast and quality of image for processing. Then, the method extracts all the boundary pixels for red lesions and blood vessels through the intensity changes analysis of image in 45 degrees and 135 degrees directions. In order to distinguish boundary pixels of red-lesions from blood vessels, a distinguishing feature for them is utilized. To extract a distinguishing feature for the boundary pixels of red-lesions, the patches around the boundary pixels are considered and the darkness and lightness of such patches is determined. For the boundary pixels of red-lesions, the nearest patch is dark and the other patches are light in all directions. In addition, in the post-processing step, we can localize the optic disc zone and remove the boundary pixels inside the zone. The reason is that there is usually no red-lesion inside optic disc zone. The proposed method has been evaluated for three datasets including Diaretdb0, Diaretdb1 and Kaggle. The new method is capable of providing 0.87, 0.89 and 0.82 for the sensitivity in Diaretdb1, Diaretdb0, and Kaggle datasets, respectively. Also, the values of 0.88, 0.9 and 0.9 are provided by the proposed method for the specificity in Diaretdb1, Diaretdb0, and Kaggle datasets, respectively. The evaluations of the proposed method show that it has improved performance compared to the related existing works.

## Supplementary Information

Supplementary Information.
