# Supplementary material for: Red-lesion extraction in retinal fundus images by directional intensity changes’ analysis
Source: Sci Rep. 2021 Sep 14;11:18223. doi: 10.1038/s41598-021-97649-x (PMC8440775; doi:10.1038/s41598-021-97649-x)
Supplement: Supplementary file 1 — Supplementary Information. [file 41598_2021_97649_MOESM1_ESM.docx]

**Supplementary Information**

**Title of article:** Red-lesion Extraction in Retinal Fundus Images by Directional Intensity Changes’ Analysis

**Authors:** Maryam Monemian and Hossein Rabbani

**Corresponding author:** Hossein Rabbani, Medical Image & Signal Processing Research Center, School of Advanced Technologies in Medicine, Isfahan University of Medical Sciences, Isfahan, Iran, 8174673461, email: Rabbani.h@gmail.com

**Table S1.** The summary of datasets, sensitivity and specificity for the related existing methods.

| Method reference | dataset | sensitivity | specificity |
| --- | --- | --- | --- |
| 17 | Utrecht | 1 | 0.87 |
| 26 | Diaretdb0, Diaretdb1 | 0.99 for Diaretdb0, 0.99 for Diaretdb1 | 0.99 for Diaretdb0, 0.99 for Diaretdb1 |
| 35 | Diaretdb1 | 0.88 | 0.91 |
| 32 | Time angiographs, GFC photographs | 0.9 for GFC photographs, 0.94 for time angiographs | Not presented |
| 36 | e-ophtha, ROC | 0.77 for ROC, 0.77 for e-ophtha | Not presented |
| 38 | e-ophtha, ROC | 0.38 for ROC, 0.57 for e-ophtha | Not presented |
| 39 | ROC | 0.62 | Not presented |
| 43 | Messidor, Kaggle | 0.84 for Kaggle, 0.9 for Messidor | 0.85 for Kaggle, 0.9 for Messidor |


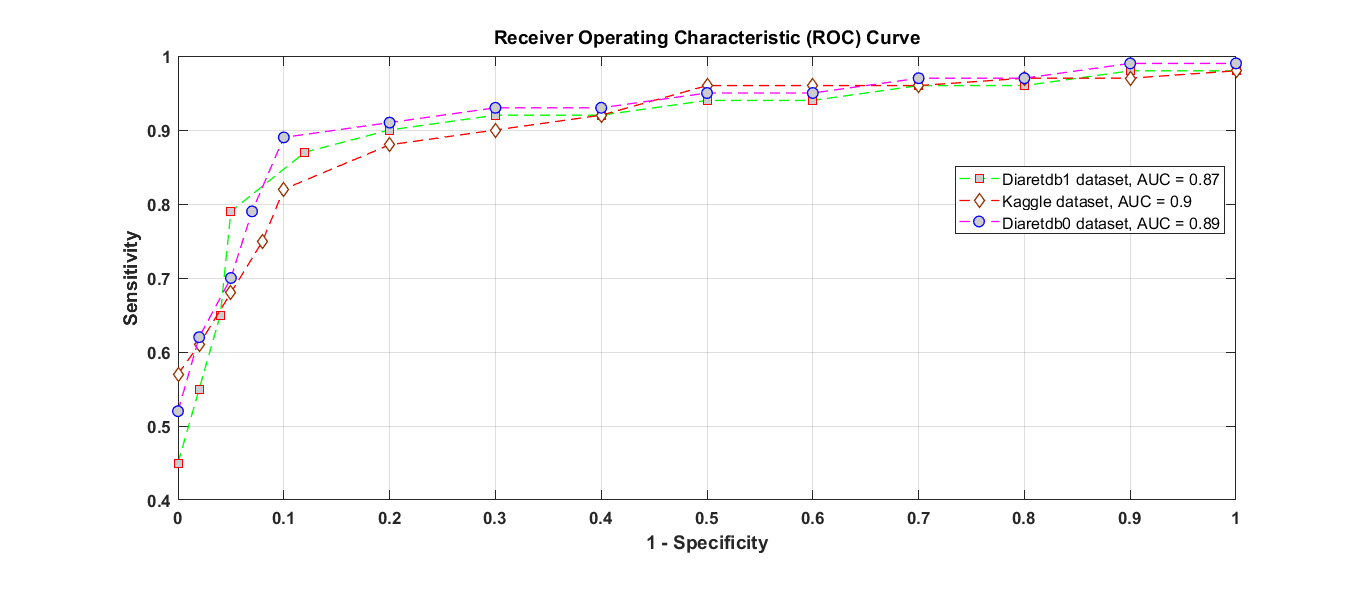


**Fig. S1.** ROC curves of REDICA for Diaretdb0, Diaretdb1 and Kaggle datasets.
